# The Effects of Antipsychotics on the Synaptic Plasticity Gene *Homer1a* Depend on a Combination of Their Receptor Profile, Dose, Duration of Treatment, and Brain Regions Targeted

**DOI:** 10.3390/ijms21155555

**Published:** 2020-08-03

**Authors:** Felice Iasevoli, Elisabetta Filomena Buonaguro, Camilla Avagliano, Annarita Barone, Anna Eramo, Licia Vellucci, Andrea de Bartolomeis

**Affiliations:** 1 Laboratory of Molecular and Translational Psychiatry and Unit of Treatment Resistant Psychosis, Section of Psychiatry, Department of Neuroscience, Reproductive Science, and Odontostomatology, University of Naples Federico II, 80131 Napoli, Italy; felice.iasevoli@unina.it (F.I.); lisabuonaguro@hotmail.com (E.F.B.); camilla.avagliano@gmail.com (C.A.); annaritabarone1@gmail.com (A.B.); liciavellucci2@gmail.com (L.V.); 2 Lundbeck LLC, Deerfield, IL 60015, USA; AERA@Lundbeck.com

**Keywords:** psychosis, synaptic plasticity, gene expression, dopamine, glutamate, haloperidol, asenapine, olanzapine, treatment resistant schizophrenia

## Abstract

Background: Antipsychotic agents modulate key molecules of the postsynaptic density (PSD), including the *Homer1a* gene, implicated in dendritic spine architecture. How the antipsychotic receptor profile, dose, and duration of administration may influence synaptic plasticity and the *Homer1a* pattern of expression is yet to be determined. Methods: In situ hybridization for *Homer1a* was performed on rat tissue sections from cortical and striatal regions of interest (ROI) after acute or chronic administration of three antipsychotics with divergent receptor profile: Haloperidol, asenapine, and olanzapine. Univariate and multivariate analyses of the effects of topography, treatment, dose, and duration of antipsychotic administration were performed. Results: All acute treatment regimens were found to induce a consistently higher expression of *Homer1a* compared to chronic ones. Haloperidol increased *Homer1a* expression compared to olanzapine in striatum at the acute time-point. A dose effect was also observed for acute administration of haloperidol. Conclusions: Biological effects of antipsychotics on *Homer1a* varied strongly depending on the combination of their receptor profile, dose, duration of administration, and throughout the different brain regions. These molecular data may have translational valence and may reflect behavioral sensitization/tolerance phenomena observed with prolonged antipsychotics.

## 1. Introduction

Antipsychotics are considered the gold-standard treatment for psychosis [1]. Despite the well-known involvement of dopamine D2 receptor (D2R) occupancy in the antipsychotic mechanism of action [2,3], and the demonstrated effects on synaptic plasticity and metaplasticity [4], there is still a dearth of information on some basic aspects regarding the antipsychotic impact on the synapse. How antipsychotic dose, receptor profile, duration of administration, and brain region targeted by each agent may influence synaptic plasticity is yet to be determined. A better understanding of these variables in antipsychotic effects may also influence our knowledge of the mechanism responsible for the loss or reduction of efficacy over time of antipsychotics treatment.

Notably, antipsychotic agents have been found to modulate the expression of glutamatergic genes of the postsynaptic density (PSD), an electron-dense thickening at the surface of the glutamatergic postsynaptic membrane [5,6,7], actively involved in behavioral disorders’ molecular pathophysiology [8,9]. Among the most replicated findings, antipsychotics have been found to modulate the immediate-early gene *Homer1a* [10,11,12], whose expression has been demonstrated to sculpt the dendritic spine and overall synaptic plasticity [5,13].

Moreover, Homer has been per se considered a candidate gene in schizophrenia and other psychiatric diseases [14,15,16,17,18] and has been demonstrated to be involved in animal models of behavioral disorders, including schizophrenia [19,20,21,22,23,24,25]. The *Homer1a* gene codes for the truncated isoform of the long Homer1 protein. Once expressed, *Homer1a* competes with the long isoforms and disassembles protein clusters formed by the long Homer isoforms and multiple PSD targets, including PSD-95, Shank, and mGluR5 [26]. As a result of *Homer1a* induction, the synaptic microdomain in which Homer proteins operate undergoes multiple functional and architectural rearrangements, including changes in intracellular Ca^++^ dynamics [27], shifts in surface channel functioning [28], and modifications in synaptic ultrastructure [29]. It modulates the interaction between metabotropic glutamatergic receptors (mGluRs) and intracellular effectors [30], thereby modulating the strength and direction of intracellular signaling [31]. Changes in Homer1a levels within the PSD may, in turn, affect the reciprocal interaction between NMDA and mGluRs, and regulate surface channel activity [28,32]. Given the complexity of Homer1a biological action, which is intrinsic to synaptic plasticity, the antipsychotic liability of modulating the expression of this gene appears relevant to these agents’ molecular actions.

In previous reports, indirect and only partially informative evaluations of outcomes of the divergent duration of treatments (i.e., acute vs. chronic effects) on *Homer1a* expression were provided [33,34,35]. A direct and systematic evaluation of the combined effects of the dose and receptor profile has not been carried out yet. Based on all these considerations, we wanted to test the hypothesis that the putative effects of antipsychotics on the *Homer1a* gene expression dose (or antipsychotic receptor profile) may be differentially mediated by the dose, receptor profile, duration of treatment, and regions of interest at which the gene expression was evaluated.

With the scope of tackling these issues, univariate analyses were run to assess independent time, dose, or receptor profile effects within each region of interest (ROI). Furthermore, as a secondary goal, multivariate analyses were run to study the patterns of *Homer1a* mRNA expression throughout multiple cortical and striatal regions of interest after acute (i.e., single administration) or chronic (21-day administration period) treatment regimens.

The dose effect was investigated by incremental doses of the high multi-receptor second-generation antipsychotic asenapine, characterized by the breadth of activities at multiple serotonergic receptors [36,37]. To investigate the dose effect by a first-generation narrower receptor profile agent, in a separate set of analyses, we compared gene expression by three different doses of the relatively selective D2R blocker haloperidol. In the third set of analysis, we investigated the receptor profile effect on gene expression by comparing asenapine, haloperidol, and olanzapine at behaviorally and neurochemically comparable doses. Olanzapine was chosen as a reference second-generation antipsychotic, with a receptor profile and biological effects that may be considered sharply divergent from asenapine [38].

Interestingly, these three compounds exhibit significantly different D2/D1 ratios (25:1 for haloperidol, 3:1 for olanzapine, and approximately 1:1 for asenapine) [38,39,40] and this feature may be relevant in order to understand the contribution of the dopaminergic antagonism in triggering the transcription of key molecules of synaptic architecture, such as Homer1a. In fact, as well as D2R-blockers, even selective D1R antagonists have been found to influence striatal *Homer1a* expression considerably [41]. Moreover, asenapine acts as a more potent antagonist in particular at 5-HT1A and 5-HT2 receptors (pKB = 7.4 for 5-HT1A; pKB = 9 for 5-HT2), in comparison to olanzapine (pKB < 5 for both 5-HT1A and 5-HT2) [38]. Testing an antipsychotic agent with such remarkable serotonergic action was even more appealing for their potential to induce cortical *Homer1a* expression [35]. Therefore, although olanzapine and asenapine both belong to the class of atypical agents and show a complex, partially overlapping, multireceptor binding profile, they may widely differ in terms of glutamatergic activity and capability to affect synaptic plasticity.

## 2. Results

A three-way mixed ANOVA was run to understand the effects of ROI, treatment, and time on *Homer1a* mRNA expression. Data are the mean fold changes of the relative disintegration per minutes (DPM) compared to the vehicle ± standard error mean. Shapiro–Wilk’s test confirmed that the relative DPM values were normally distributed (Tables S1–S6). Levene’s test for equality of variances showed homogeneity of variances for the relative DPM throughout all ROIs. Outputs using raw (i.e., non-relativized) data are reported in Tables S7–S10.

### 2.1. Paradigm 1

**Figure 1:**
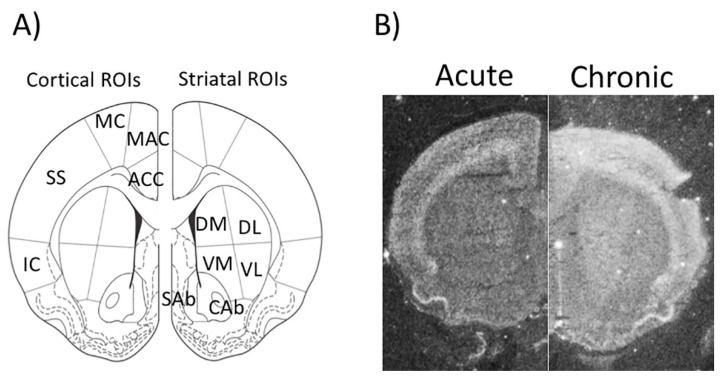
(**A**) Panel A: diagrams of regions of interest (ROIs) quantitated on autoradiographic film images in the rat forebrain in which gene expression was measured. ACC = anterior cingulated cortex; MAC = medial agranular cortex; MC = motor cortex; SS = somatosensory cortex; IC = insular cortex; DM = dorso-medial caudate putamen; DL = dorso-lateral caudate putamen; VM = ventro-medial caudate putamen; VL = ventro-lateral caudate putamen; Cab = core of the nucleus accumbens; Sab = shell of the nucleus accumbens. Modified from Paxinos and Watson (1997); (**B**) Panel B: representative juxtaposed autoradiogram sections from short- (left side) and long-term (right side) treatment by vehicle.

In this paradigm, the three categorical independent variables were: Treatment with incremental asenapine doses (thereafter asenapine Doses); time; and ROIs. The asenapine doses variable comprised three categories: 0.05 mg/kg treatment group; 0.1 mg/kg treatment group; and 0.3 mg/kg treatment group. The time variable comprised two categories: Acute administration and chronic administration. The ROIs variable comprised five categories in the cortex (anterior cingulate cortex, ACC; medial agranular cortex, MAC; motor cortex, MC; somatosensory cortex, SS; insular cortex, IC) and six categories in striatum (dorso-medial caudate putamen, DM; dorso-lateral caudate putamen, DL; ventro-medial caudate putamen, VM; ventro-lateral caudate putamen, VL; nucleus accumbens core, CAb; nucleus accumbens shell, Sab; Figure 1).

#### 2.1.1. Striatum

**Figure 2:**
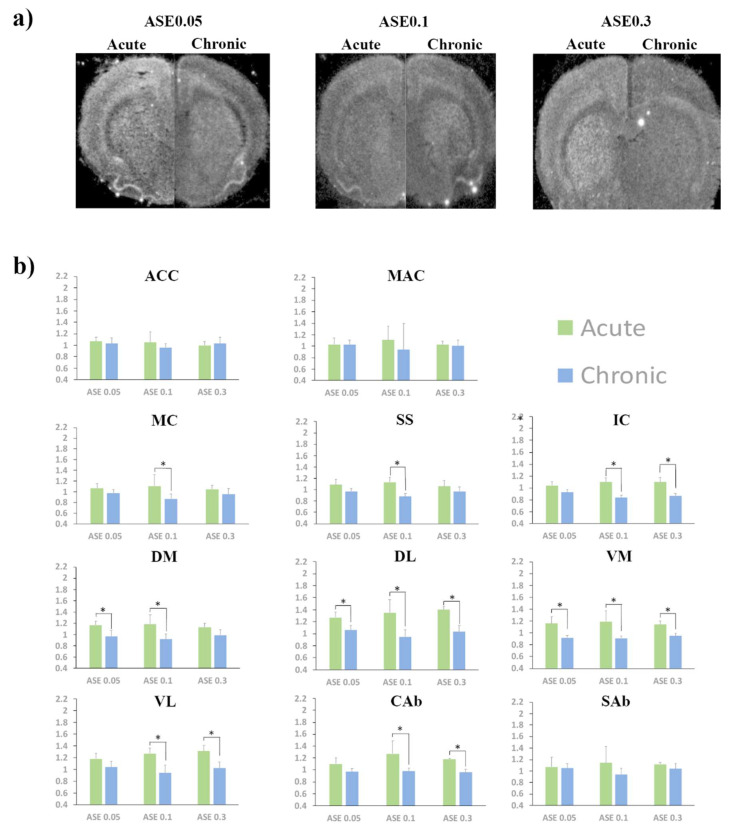
(**a**) Upper section: representative juxtaposed autoradiogram sections from acute (left side) and chronic (right side) treatment by asenapine at 0.05 mg/kg (ASE 0.05), 0.1 mg/kg (ASE 0.1), and 0.3 mg/kg (ASE 0.3), respectively; (**b**) Lower section: levels of *Homer1a* mRNA expression by different doses of asenapine at either acute (green bars) or chronic (blue bars) time points in the cortical and striatal regions of interest (ROIs). Gene expression is given as fold changes of vehicle-induced relative DPMs ± standard error mean (S.E.M.). ASE0.1 = asenapine at 0.1 mg/kg; ASE0.3 = asenapine at 0.3 mg/kg; ACC = anterior cingulated cortex; MAC = medial agranular cortex; MC = motor cortex; SS = somatosensory cortex; IC = insular cortex; DM = dorso-medial caudate putamen; DL = dorso-lateral caudate putamen; VM = ventro-medial caudate putamen; VL= ventro-lateral caudate putamen; Cab = core of the nucleus accumbens; Sab = shell of the nucleus accumbens. *: significantly different groups at Student’s t test (time effect). Significantly different groups are connected by brackets.

No significant effect of asenapine doses was found in any striatal ROI at either acute or chronic time point (Table 1, Figure 2).

A statistically significant effect of time was found for all doses in distinct striatal ROIs (Table 1, Figure 2). More specifically, mean *Homer1a* expression was higher after acute compared to chronic administration of: ASE0.05 in DM, DL, and VM; ASE0.1 in all striatal ROIs with the exception of SAb; and ASE0.3 in DL, VM, VL, and CAb (Table 1, Figure 2).

At the multivariate analysis, we found a statistically significant interaction effect between time, asenapine dose, and ROI (Table 4). Significant ROI*time and ROI*asenapine dose interactions were subsequently found, while no asenapine dose*time interaction was observed (Table 4).

#### 2.1.2. Cortex

At both the acute and chronic time-points, no significant asenapine dose effect was found in cortical ROIs (Table 1, Figure 2).

A statistically significant effect of time was found in multiple cortical ROIs (Table 1). *Homer1a* expression was significantly higher after acute compared to chronic administration of ASE0.1 in MC, SS, and IC and of ASE0.3 in IC (Table 1, Figure 2).

At the multivariate analysis, no significant three-way interaction between ROI, asenapine dose, and time was found (Table 4). A significant ROI*time two-way interaction was found, while no significant asenapine dose*ROI and asenapine dose*time two-way interactions were observed (Table 4).

### 2.2. Paradigm 2

In this paradigm, the three categorical independent variables were: Treatment with incremental haloperidol doses (thereafter haloperidol doses); time; and ROIs. The haloperidol doses variable comprised three categories: 0.25 mg/kg treatment group; 0.5 mg/kg treatment group; and 0.8 mg/kg treatment group. The time variable comprised two categories: Acute administration and chronic administration. The ROIs variable comprised five categories in the cortex (ACC; MAC; MC; SS; IC) and six categories in the striatum (DM; DL; VM; VL; CAb; SAb).

#### 2.2.1. Striatum

**Figure 3:**
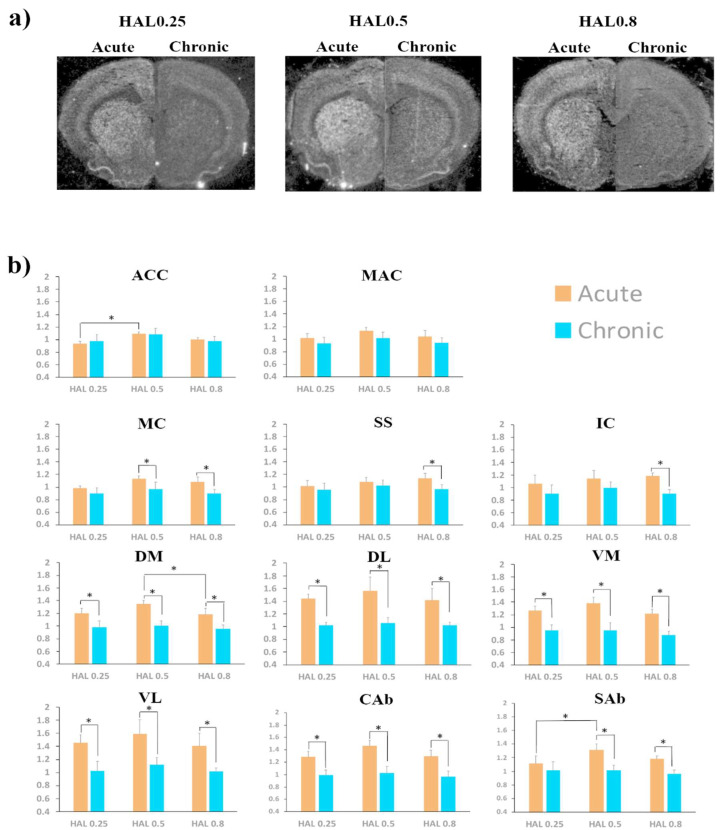
(**a**) Upper section: representative juxtaposed autoradiogram sections from acute (left side) and chronic (right side) treatment by haloperidol at 0.25 mg/kg (HAL 0.25), 0.5 mg/kg (HAL 0.5), and 0.8 mg/kg (HAL 0.8), respectively; (**b**) Lower section: levels of *Homer1a* mRNA expression by different doses of haloperidol at either acute (orange bars) or chronic (turkey bars) time points in the cortical and striatal regions of interest (ROIs). Gene expression is given as fold changes of vehicle-induced relative DPMs ± standard error mean (S.E.M.). HAL0.25 = haloperidol at 0.25 mg/kg; HAL0.5 = haloperidol at 0.5 mg/kg; HAL0.8 = haloperidol at 0.8 mg/kg; ACC = anterior cingulated cortex; MAC = medial agranular cortex; MC = motor cortex; SS = somatosensory cortex; IC = insular cortex; DM = dorso-medial caudate putamen; DL = dorso-lateral caudate putamen; VM = ventro-medial caudate putamen; VL = ventro-lateral caudate putamen; Cab = core of the nucleus accumbens; Sab = shell of the nucleus accumbens; *: significantly different groups at Bonferroni’s post-hoc test (haloperidol dose effect) or at Student’s t test (time effect). Significantly different groups are connected by brackets.

A significant haloperidol dose effect was observed in the DM and the SAb after acute administration (Table 2, Figure 3). In the DM, *Homer1a* expression by HAL0.5 was significantly higher than expression by HAL0.8 (Bonferroni adjusted post-hoc test: *p* = 0.05; Figure 3). In the SAb, HAL0.5 administrations triggered significantly higher expression of *Homer1a* than HAL0.25 (Bonferroni adjusted post-hoc test: *p* = 0.032; Figure 3). At the chronic time-point, no significant dose effect was found in any ROI (Table 2, Figure 3).

A significant time effect was found in all striatal ROIs (Table 2). Specifically, in all ROIs, acute expression of *Homer1a* was significantly higher than chronic expression by HAL0.25, HAL0.5, and HAL0.8, with the only exception of the SAb for the HAL0.25 dose (Table 2, Figure 3).

At the multivariate analysis, we did not find a significant three-way interaction effect between ROI, haloperidol dose, and time (Table 4). A significant ROI*time two-way interaction was found, while no significant haloperidol dose*ROI and haloperidol dose*time two-way interactions were observed (Table 4).

#### 2.2.2. Cortex

A significant haloperidol dose effect was found in the ACC at the acute time-point (Table 2). In this region, *Homer1a* expression by HAL0.5 was significantly higher than expression by HAL0.25 (Bonferroni adjusted post-hoc test: *p* = 0.027; Figure 3). At the chronic time-point, no significant haloperidol dose effect was found (Table 2, Figure 3).

A significant time effect was found in the MC, SS, and IC regions (Table 2). In all these regions, *Homer1a* expression by HAL0.8 was significantly higher after the acute than the chronic administration regimen (Figure 3). In the MC, *Homer1a* expression by acute HAL0.5 was significantly higher than by chronic HAL0.5 administration (Table 2, Figure 3).

At the multivariate analysis, no significant three-way interaction between ROI, haloperidol dose, and time was observed (Table 4). A significant ROI*time two-way interaction was found, while no significant haloperidol dose*ROI and haloperidol dose*time two-way interactions were observed (Table 4).

### 2.3. Paradigm 3

In this paradigm, the three categorical independent variables were: Treatment with antipsychotics with different receptor profiles (thereafter treatment); time; and ROIs. The treatment variable comprised three categories: Olanzapine 2.5 mg/kg treatment group; haloperidol 0.5 mg/kg treatment group; and asenapine 0.1 mg/kg treatment group. The time variable comprised two categories: Acute administration and chronic administration. The ROIs variable comprised five categories in the cortex (ACC; MAC; MC; SS; IC) and six categories in the striatum (DM; DL; VM; VL; CAb; SAb).

The choice of antipsychotic doses to be compared was based on multiple considerations. These doses were reported to elicit a striatal D2 receptor occupancy ranging between 80% and 90% [42], and to exert comparable effects in both behavioral tasks relevant to antipsychotic action [43,44] and studies evaluating neurophysiological and neurochemical outcomes [45,46,47].

#### 2.3.1. Striatum

**Figure 4:**
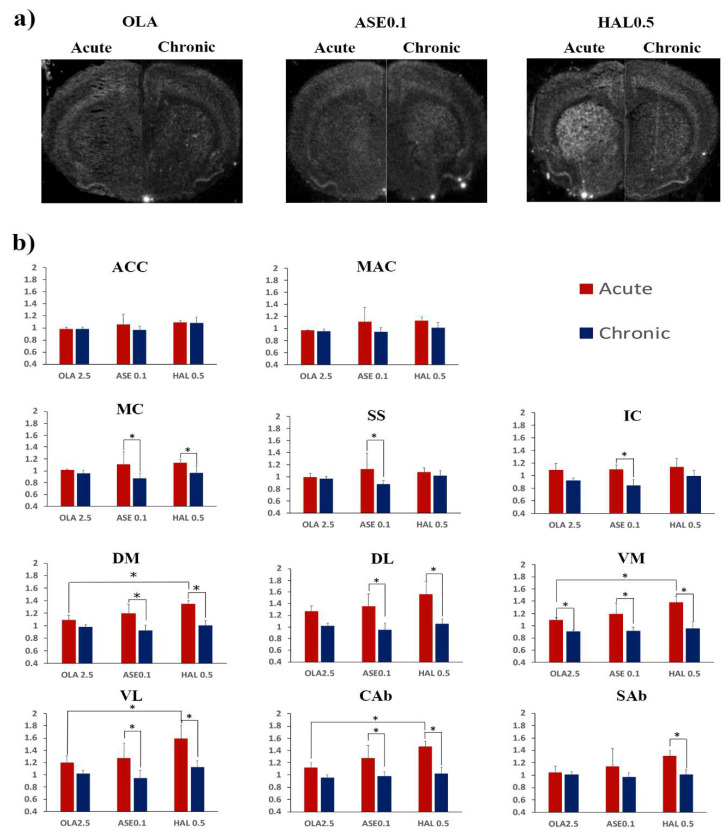
(**a**) Upper section: representative juxtaposed autoradiogram sections from acute (left side) and chronic (right side) treatment by olanzapine (OLA2.5), asenapine 0.1 mg/kg (ASE 0.1), and haloperidol 0.5 mg/kg (HAL 0.5), respectively; (**b**) Lower section: levels of *Homer1a* mRNA expression by olanzapine, asenapine, and haloperidol at either acute (red bars) or chronic (dark blue bars) time points in the cortical and striatal ROIs. Gene expression is given as fold changes of vehicle-induced relative DPMs ± standard error mean (S.E.M.). OLA2.5 = olanzapine at 2.5 mg/kg; ASE0.1 = asenapine at 0.1 mg/kg; HAL0.5 = haloperidol at 0.5 mg/kg; ACC = anterior cingulated cortex; MAC = medial agranular cortex; MC = motor cortex; SS = somatosensory cortex; IC = insular cortex; DM = dorso-medial caudate putamen; DL = dorso-lateral caudate putamen; VM = ventro-medial caudate putamen; VL = ventro-lateral caudate putamen; Cab = core of the nucleus accumbens; Sab = shell of the nucleus accumbens. *: significantly different groups at Bonferroni’s post-hoc test (treatment effect) or at Student’s t test (time effect). Significantly different groups are connected by brackets.

A significant treatment effect was found in the DM, VM, VL, and CAb regions at the acute time point (Table 3). In all these regions, HAL0.5 triggered significantly higher *Homer1a* expression than OLA2.5 (Bonferroni adjusted post-hoc test: DM, *p* = 0.016; VM, *p* = 0.014; VL, *p* = 0.031; CAb, *p* = 0.013; Figure 4). No significant treatment effect was observed after chronic antipsychotic administration (Table 3).

A significant time effect was found in all striatal ROIs (Table 3). Acute HAL0.5 and ASE0.1 induced higher *Homer1a* expression than chronic HAL0.5 and ASE0.1, respectively, in all striatal regions, with the exception of the SAb for ASE0.1 (Figure 4). Acute OLA2.5 triggered higher *Homer1a* expression than chronic OLA2.5 in the VM (Figure 4).

At the multivariate analysis, no significant three-way interaction between ROI, treatment, and time was observed (Table 4). A significant ROI*time two-way interaction was found, while no significant treatment*ROI and treatment*time two-way interactions were observed (Table 4).

#### 2.3.2. Cortex

No significant treatment effect was observed at either the acute or the chronic time-point (Table 3).

A significant time effect was found in the MC, SS, and IC regions (Table 3). In all these regions, ASE0.1 acute administration induced significantly higher *Homer1a* expression than ASE0.1 chronic administration (Figure 4). In the MC, acute HAL0.5 also induced significantly higher *Homer1a* expression than chronic HAL0.5 (Figure 4).

At the multivariate analysis, no significant three-way interaction between ROI, treatment, and time was observed (Table 4). A significant ROI*time two-way interaction was found, while no significant treatment*ROI and treatment*time two-way interactions were observed (Table 4).

## 3. Discussion

**Figure 5:**
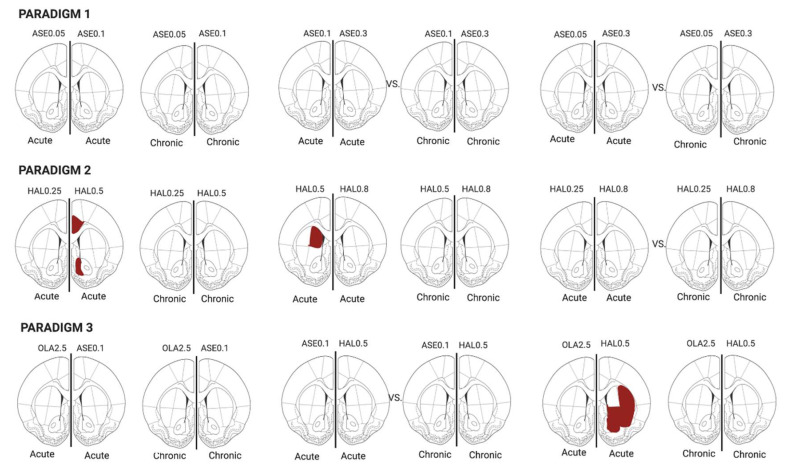
The treatment effect was analyzed for each region of interest (ROI) at both acute and chronic time-points. The red-colored ROI are those expressing significantly higher *Homer1a* levels compared to the corresponding ROI at the other treatment regimen. VEH= vehicle; ASE0.05 = asenapine 0.05 mg/kg; ASE0.1 = asenapine 0.1 mg/kg; ASE0.3 = asenapine 0.3 mg/kg; HAL0.25= haloperidol 0.25 mg/kg; HAL0.5 = haloperidol 0.5 mg/kg; HAL0.8 = haloperidol 0.8 mg/kg; OLA2.5 = olanzapine 2.5 mg/kg.

**Figure 6:**
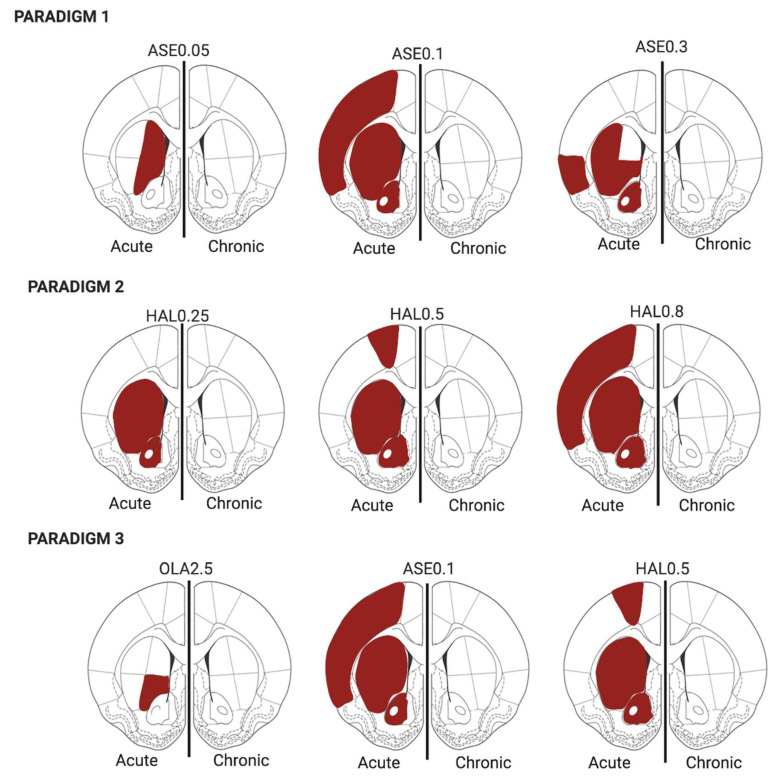
The time effect was analyzed separately for each antipsychotic treatment. The red-colored regions of interest (ROI) were those expressing significantly higher levels of *Homer1a* in comparison to the corresponding ROI at the other time-point. VEH = vehicle; ASE0.05 = asenapine 0.05 mg/kg; ASE0.1 = asenapine 0.1 mg/kg; ASE0.3 = asenapine 0.3 mg/kg; HAL0.25 = haloperidol 0.25 mg/kg; HAL0.5 = haloperidol 0.5 mg/kg; HAL0.8 = haloperidol 0.8 mg/kg; OLA2.5 = olanzapine 2.5 mg/kg.

In this work, we aimed to evaluate whether the molecular effects exerted by antipsychotics on the *Homer1a* gene, which is a marker of glutamatergic synaptic plasticity and dopamine–glutamate interaction, were modulated by the antipsychotic dose/receptor profile, the duration of administration (acute vs. repeated), and brain regions evaluated. A graphical summarization of the major results of this study is given in Figure 5 and Figure 6.

Among the major findings of this work, we found: (i) Significant treatment effects, since *Homer1a* expression was different in the selected areas and time-points among antipsychotics with different receptor profiles; (ii) significant dose effects for haloperidol in selected striatal and cortical ROIs at the acute but not the chronic time-point; (iii) significant time effects since *Homer1a* expression by all antipsychotics was significantly higher after acute administration compared to a prolonged treatment regimen; and (iv) a significant three-way interaction between topography (i.e., ROIs), antipsychotic dose, and time for asenapine in the striatum, and a significant two-way interaction between ROI and time in all groups investigated.

These data indicate that the effects of the duration of antipsychotic treatment on gene expression were affected by the brain regions where gene expression was measured. Moreover, the extent of gene expression, at least by the antipsychotics tested herein, was strongly affected by the duration of treatment, with acute (i.e., single administration) regimens inducing significantly higher *Homer1a* expression than chronic ones (i.e., 21-day-long administration period).

The data reinforced the suggestion that the biological effects of antipsychotics on *Homer1a* depend on a complex combination of the receptor profile, topography, and duration of exposure to these agents. Since Homer is a family of molecules that are crucially implicated in glutamatergic signaling, PSD architecture, and whose expression is profoundly affected by dopaminergic modulation, the evaluation of the present results may take into consideration these biological elements. Antipsychotic-mediated modulation of dopaminergic and possibly non-dopaminergic (including glutamatergic) receptors should be accounted for in mechanistic explanations of changes in *Homer1a* expression. Homer effects on glutamatergic signaling and synaptic rearrangements should be considered when discussing the antipsychotic-mediated modulation of *Homer*.

As expected, the first-generation antipsychotic haloperidol induced higher *Homer1a* expression than olanzapine at the acute time-point in striatal regions. This result is consistent with the hypothesis that antipsychotic-induced *Homer1a* expression in the striatum mainly depends on the degree of the dopamine D2R antagonism, whereby acute administration of potent D2R-blockers induces greater *Homer1a* expression in comparison to atypical agents in subcortical regions. Hence, acute variations in subcortical *Homer1a* mRNA have been proposed by our group as predictors of the typical or atypical character of the antipsychotic molecules [10,48]. These observations comply with previous reports [11,20,49,50]. From a clinical point of view, it may reflect the propensity of typical compounds to provoke extrapyramidal symptoms in humans, as discussed below in more detail.

It has been reported that *Homer1a* mRNA is under a prominent glutamatergic regulation since NMDAR activation upregulates gene expression [51]. Moreover, *Homer1a* expression is mediated by selective D2R antagonism [10], and possibly 5-HT2R antagonism and D1R agonism [52,53], either directly or indirectly, via glutamate-mediated pathways. Acute antipsychotic administration is known to increase, rather than decrease, extracellular levels of dopamine in the striatum, exerting a relevant blockade of presynaptic D2Rs [54,55]. Since post-synaptic D2Rs are blocked by the antipsychotic, it may be expected that dopamine may interact with post-synaptic D1Rs. In turn, activated D1Rs may cooperate with NMDARs to intensify NMDAR-mediated glutamatergic transmission [56], leading to increased *Homer1a* expression. According to these mechanistic considerations, the higher acute *Homer1a* expression by haloperidol compared to olanzapine appears to indicate a larger action on D2R by haloperidol than olanzapine, which in turn may be responsible for larger presynaptic dopamine release and larger NMDA receptor activation via post-synaptic D1Rs. Although no significant differences between haloperidol and asenapine treatments were found in this study, this may depend on the highly conservative type of post-hoc test we adopted herein, i.e., the Bonferroni correction. Notably, in a previous study [3], we found significant differences between these two agents, corroborating the view above.

A dose effect was found by haloperidol but not by asenapine. Moreover, the dose effect was demonstrated only in selected cortical and striatal areas and only after an acute treatment. Since increased *Homer1a* expression has been consistently associated to enhanced glutamatergic excitatory activity, possibly as a feedback mechanism to prevent NMDAR-mediated neuronal injury [57,58,59], our results may indicate that, in selected brain regions, acute haloperidol may trigger higher activation of glutamatergic neurons at the intermediate 0.5-mg compared to the high 0.8-mg dose.

At the 0.8-mg high dose, haloperidol may over-saturate synaptic D2Rs and interact with a fraction of D1Rs as well, competing with synaptic dopamine and therefore partially blocking intracellular transduction from these receptors. Antagonism at D1Rs would, in turn, trigger less NMDAR activation as compared to the activation given by the 0.5-mg dose. As a consequence of this reduced NMDAR activation, less *Homer1a* mRNA would be produced by neurons compared to the intermediate 0.5-mg dose. Indeed, it has been described that more than 90% striatal D2Rs are blocked by haloperidol at doses comparable to those used herein [60,61] and that haloperidol has some affinity to D1Rs, although lower than to D2Rs [62].

The loss of this pattern in chronic treatments suggests that these dynamics may be restricted to acute treatments, while more complex molecular rearrangements may occur during sustained antipsychotics, as it will be discussed below. Repeated administration may allow reaching plasma steady-state and targeting high-level D2Rs, thereby smoothing differences among doses. It has been described, by in vivo recordings of striatal D2R occupancy, that 7-day treatment with 0.25 or 0.5 mg/kg haloperidol resulted in non-significant differences in D2R occupancy, which was higher than 65% [60]. Additionally, behavioral outcomes, such as vacuous chewing movements, were found to be non-significantly different among haloperidol doses reminiscent of those used herein given for eight weeks [63]. Therefore, these studies support the view that the effects of divergent haloperidol doses mitigate during repeated administration and agree with the molecular observations in the present study.

The lack of dose dependence by asenapine may be due to the almost equivalent affinity to D1Rs and D2S by this agent or its strong serotonergic action [36,37], which is predicted to affect dopamine release and may prevent the saturation of D2Rs.

It could not be excluded that other receptor systems may intervene in these dynamics, e.g., the sigma1 receptor for haloperidol or the histaminergic, cholinergic, or serotonergic receptors for asenapine. More studies are needed to understand whether this different pattern is specific to relatively selective D2Rs blockers, such as haloperidol, and are avoided in antipsychotics with a broader receptor profile.

A consistent time effect was found for all antipsychotics tested. Accordingly, the biological effects exerted by haloperidol, asenapine, and olanzapine on *Homer1a* were significantly higher after a single acute administration than after repeated dosing. These results may indicate that antipsychotic effects on the *Homer1a*-mediated biological system diminish over repeated administrations, although these outcomes vary depending on the antipsychotic dose and brain region.

One possible explanation for these results is that prolonged antipsychotic treatment may be associated with a reduction of the depolarizing effects on neurons. Indeed, it has been demonstrated that prolonged antipsychotic treatments may cause depolarization blocks and reduce synaptic dopamine [64]. This experimental evidence may explain the loss of the dose effect observed in the present study at the chronic treatment.

Another possible explanation is an upregulation of post-synaptic D2Rs, which may cause the behavioral phenomena of sensitization/tolerance to prolonged antipsychotics [65]. The observed attenuation of *Homer1a* expression after chronic antipsychotics may represent an adaptive biological mechanism predisposing to antipsychotic sensitization/tolerance. Attenuated *Homer1a* expression may reflect (or cause) a reduction of glutamatergic neurons’ activity in targeted areas as compared to acute administration. In turn, this may depend on D2R-related (and possibly 5-HT2A receptor-related) adaptive changes, i.e., upregulation [66,67], since it has been reported that increased D2R activation reduces glutamate activation via NMDA receptors [68].

Indeed, a time effect has been reported in a behavioral task (conditioned avoidance response, CAR) after repeated haloperidol, risperidone, and olanzapine treatments [69]. Specifically, antipsychotic efficacy in suppressing avoidance response had an early onset and increased progressively [69]. On the other hand, our data showed that antipsychotic effects on *Homer1a* had an early onset but significantly declined over time. Whether these molecular changes may underlie the reported behavioral sensitization effects of antipsychotics in rodents is still challenging.

Notably, *Homer1a* overexpression in striatal sites has been associated with reduced locomotor activity [70]. Remarkably, glutamatergic hyperactivity in the striatum has been considered a major pathophysiological mechanism in Parkinson’s disease as well as in L-DOPA-induced dyskinesia [71,72,73]. It may be hypothesized that increased *Homer1a* expression by acute antipsychotics may be the consequence of glutamatergic activation in the striatum, which in turn may concur to cause acute extrapyramidal effects. The relative attenuation of *Homer1a* expression by persistent antipsychotic treatment may indicate a reduced striatal glutamatergic activation, possibly as a consequence of dopamine receptor sensitization, which may lead to an attenuation of the imbalance in motor-related circuits and reduced motor side effects. This hypothesis paves the way to the possibility of manipulating *Homer1a* expression as a tool to affect motor side effects of antipsychotics. Nonetheless, this hypothesis should be tested in specifically designed experiments.

Significant acute vs. chronic changes were found in the insular cortex with haloperidol and asenapine. From a neurobiological and neuropharmacological point of view, these results may implicate that the insular cortex represents a preferential site in which antipsychotics exert time-dependent effects, irrespective of the antipsychotic used. From a clinical point of view, the insular cortex has been considered a crucial region for the salience network, which is widely affected by dopaminergic and glutamatergic neurotransmission, and is involved in the ability to discriminate between self-generated and external information [74,75]. Alterations in the salience network and insula overactivations may underlie many of the clinical features of psychotic disorders [76,77]. Moreover, antipsychotic treatments have been found to restore insular abnormalities in patterns of connectivity [78]. In this context, decreased *Homer1a* expression after chronic antipsychotic exposure in this peculiar region may possibly indicate a reduced glutamatergic activation exerted by neuroleptics, which in turn may underpin potential mitigating effects on abnormal salience signaling.

One challenging result of our study is that despite evidence that topography, treatment, and duration of antipsychotic administration may combine to determine multiple differential patterns of *Homer1a* expression, multivariate analysis of the interactions among these factors was often negative. Despite the fact that the study was adequately powered to run univariate analyses, the power for multivariate analyses was lower, determining the possibility of false negative outcomes.

In conclusion, *Homer1a* gene expression is affected by antipsychotics with a specific dose, receptor profile, topography, and time-related dynamics. These data may deepen the knowledge of antipsychotics neurobiology and may have translational valence to infer their unique clinical action and side effects.

## 4. Materials and Methods

### 4.1. Animals and Drug Treatments

Male Sprague-Dawley rats, mean weight 250 g (Charles River Labs, Lecco, Italy), were obtained, and housed in a colony room at controlled temperature and humidity, under a 12 h light/dark cycle, with ad libitum access to laboratory chow and water. In the first days, animals were adapted to human handling. All procedures were approved by the local Animal Care and Use Committee and were in accordance to the NIH Guide for Care and Use of Laboratory Animals (NIH Publication No. 85-23, revised 1996). We used the least number of animals possible to obtain reliable results.

### 4.2. Study Design and Drug Treatment

The following agents were used: (1) Asenapine maleate (supplied as a powder by H. Lundbeck A/S, Copenhagen, Denmark), olanzapine, and haloperidol (both supplied as a powder by Sigma-Aldrich, Milan, Italy). The powder was dissolved in saline solution (NaCl 0.9%), adjusted to physiological pH, and injected i.p. (final volume: 1 mL/kg).

A total of 80 animals were used for the experimental procedures. Rats were randomly assigned to one of the following treatments (*n* = 10 for each): Vehicle (NaCl 0.9%, VEH); asenapine 0.05 mg/kg (ASE0.05); asenapine 0.1 mg/kg (ASE0.1); asenapine 0.3 mg/kg (ASE0.3); haloperidol 0.25 mg/kg (HAL0.25); haloperidol 0.5 mg/kg (HAL0.5); haloperidol 0.8 mg/kg (HAL0.8); and olanzapine 2.5 mg/kg (OLA2.5). All drugs were administered at doses that were reported to be behaviorally active in previous works [3,12,35,38,49,79,80]. The doses of asenapine and haloperidol were in the dose range that has been used in multiple experimental paradigms to mimic the neurochemical and behavioral effects of antipsychotics [42,47,61,81,82,83,84].

Rats were then split into two sets: Those sacrificed 90 min after single drug injection (i.e., acute dose regimen), and those sacrificed 90 min after the last administration of a chronic dose regimen (i.e., 21-day consecutive treatment). As a result, rats were ultimately subdivided in the following groups (*n* = 5 each): Acute VEH; chronic VEH; acute ASE0.05; chronic ASE0.05; acute ASE0.1; chronic ASE0.1; acute ASE0.3; chronic ASE0.3; acute HAL0.25; chronic HAL0.25; acute HAL0.5; chronic HAL0.5; acute HAL0.8; chronic HAL0.8; acute OLA2.5; and chronic OLA2.5.

### 4.3. In Situ Hybridization

After sacrifice, animal brains were frozen on powdered dry ice and stored at −70 °C. Sectioning was made on a cryostat at −18 °C. Serial coronal sections of 12 μm were obtained at the level of the middle-rostral striatum (approximately from Bregma 1.20 to 1.00 mm). The rat brain atlas by Paxinos and Watson [85] was used as an anatomical reference. Sections were stored at −80 °C for subsequent analysis.

We initially designed a series of probes for radioactive in situ hybridization by Gene-Bank, among those oligodeoxyribonucleotide sequences that were found complementary to the mRNA sequence of the *Homer1a* gene. The initially designed sequences were double-checked with BLAST in order to avoid cross-hybridization. The selected probe was that ensuring the best specificity/sensitivity ratio and was manufactured by MWG Biotech, Milan, Italy.

On the day of the hybridization, the sections were prepared to receive the hybridization mix by sequential washes into 4% formaldehyde, PBS (pH 7.4), 0.25% acetic anhydride in 0.1 M triethanolamine/0.9% 25 NaCl, ethanol, chloroform, again in ethanol, and finally air-dried.

The labeling reaction mix consisted of 100 mCi ^35^S-dATP, 125 units of terminal deoxynucleotidyl transferase (TdT), and 7.5 pmol/µL of oligodeoxyribonucleotide. The ProbeQuant G-50 Micro Columns (supplied by Amersham-GE Healthcare Biosciences, Italy) were used to separate unincorporated nucleotides from radiolabeled DNA.

Sections were hybridized with 0.4–0.6 × 10^6^ 2 cpm of labeling reaction mix that was buffered by 0.2 mg/mL heparin sulfate, 50% formamide, 10% dextran sulfate, 4 mM EDTA, 0.1% pyrophosphate, 600 mM NaCl, and 80 mM Tris-HCl (pH 7.5).

Hybridized sections were incubated at 37 °C in a humidified chamber for 22–24 h and subsequently washed in 2× SSC/50% formamide at 43–44 °C in 1× SSC at room temperature. After drying, sections were exposed to an autoradiographic film (Kodak-10 Biomax MR). Sections were co-exposed with a ^14^C standards’ slide (ARC-146C, American Radiolabeled Chemical). Time of exposure was set to maximize the signal-to-noise ratio.

The ImageJ software (v. 1.46v, http://rsb.info.nih.gov/ij/) was used for quantitation of the optical density of the autoradiographic signal. Brain sections from both rats treated acutely and rats treated chronically were co-exposed to the same X-ray sheet to allow comparative statistical analysis. Optical density was measured within outlined regions of interest (ROIs), in correspondence of the cortex (ACC, MAC, MC, SS, and IC), and the striatum (DM, DL, VM, VL, Cab, and Sab) (Figure 1). The ^14^C standard values from 4 through 12 were previously cross-calibrated to ^35^S brain paste standards to obtain a calibration curve based on a “best fit” 3rd-degree polynomial. The calibration curve was used to convert the measurements of the optical density into “relative DPM”.

For statistical analysis, data from each antipsychotic treatment were given as fold changes of the gene expression to the vehicle-induced expression. Specifically, acute antipsychotic signal intensity was given as fold changes of the acute vehicle signal intensity, and chronic antipsychotic signal intensity as fold changes of the chronic vehicle. The whole in situ hybridization procedure was performed blinded with coded frozen brains.

### 4.4. Statistical Analysis

The statistical analyses were performed using JMP9.0.1 and SPSS 24.0 software. The Shapiro–Wilk test was used to confirm normal distribution of the continuous variable (i.e., signal intensity of gene expression). ROIs, treatment, and time were the categorical variables that were used to analyze gene expression.

As a first goal, we wanted to capture significant effects of independent time or treatment in any topographic subdivision. Therefore, we carried out separate univariate analyses of time and treatment effect in each ROI. In all tests, significance was set at *p* < 0.05. The Bonferroni corrected post-hoc tests were used for pairwise comparisons in univariate analyses.

To contemporarily evaluate the effects of antipsychotic dose/receptor profile, topography (i.e., ROIs), and duration of treatment (i.e., time), we carried out a 3way mixed ANOVA, with ROIs as the within-subjects factor, and treatment and time as the between-subjects factors. To avoid multiple significant *p*-values of limited practical relevance, cortical and striatal ROIs were separately assessed since they represent distinct anatomical divisions within the forebrain, although functionally connected.

Follow-up evaluations of 2way interactions with the within-subjects factor (i.e., ROI*treatment; ROI*time) or the between-subjects factors only (treatment*time) were carried out.

According to the aims of this study, three separate analyses were carried out. Paradigm 1 and paradigm 2 examined whether *Homer1a* expression may be significantly different among increasing doses of a second-generation multireceptor antipsychotic (i.e., asenapine) and a first-generation antipsychotic with almost selective D2R antagonist action (i.e., haloperidol). Accordingly, *Homer1a* expression was separately compared throughout asenapine-treated animals (asenapine groups, paradigm 1) and throughout haloperidol-treated animals (Haloperidol Groups, Paradigm 2). Paradigm 3 was aimed at evaluating whether multireceptor profile and substantial action on serotonin receptors may affect the cortical or striatal expression of *Homer1a* differentially. Accordingly, *Homer1a* expression was compared among the intermediate asenapine and haloperidol doses and a behaviorally compatible olanzapine dose, a multireceptor antipsychotic with a broader receptor profile but narrower action on serotonin receptors compared to asenapine.

## Figures and Tables

**Table 1:** Dose and time-independent effects on asenapine-mediated *Homer1a* expression as assessed by univariate analysis in each region of interest. On the right side of the table, the “dose effect” was analyzed separately for acute and chronic administration regimens, in order to determine if asenapine-mediated *Homer1a* expression was different for groups exposed to different asenapine doses (i.e., ASE0.05, ASE0.1, and ASE0.3). On the left side of the table, the “time effect” was analyzed separately for each asenapine dose, in order to determine if asenapine-mediated *Homer1a* expression was different for groups with different durations of treatment (i.e., acute and chronic). Significant effects are given in bold. ASE0.05 = asenapine at 0.05 mg/kg; ASE0.1 = asenapine at 0.1 mg/kg; ASE0.3 = asenapine at 0.3 mg/kg. ns = not significant.

		Dose Effect						Time Effect			
		Acute ASE0.05 vs. Acute ASE0.1 vs. Acute ASE0.3		Chronic ASE0.05 vs. Chronic ASE0.1 vs. Chronic ASE0.3		Acute ASE0.05 vs. Chronic ASE0.05		Acute ASE0.1 vs. Chronic ASE0.1		Acute ASE0.3 vs. Chronic ASE0.3	
		F_df_	*p*	F_df_	*p*	F_df_	*p*	F_df_	*p*	F_df_	*p*
**Striatum**	**DM**	0.20_2,17_	ns	0.50_2,17_	ns	6.86_1,17_	**0.018**	12.14_1,17_	**0.003**	2.85_1,17_	ns
	**DL**	0.92_,17_	ns	0.90_2,17_	ns	5.83_1,17_	**0.027**	21.07_1,17_	**0.0005**	13.79_1,17_	**0.002**
	**VM**	0.22_2,17_	ns	1.73_2,17_	ns	11.48_1,17_	**0.003**	15.81_1,17_	**0.001**	6.55_1,17_	**0.022**
	**VL**	069._2,17_	ns	0.49_2,17_	ns	1.88_1,17_	ns	10.17_1,17_	**0.005**	6.36_1,17_	**0.022**
	**CAb**	1.88_2,17_	ns	0.03_2,17_	ns	2.36_1,17_	ns	11.32_1,17_	**0.004**	5.42_1,17_	**0.033**
	**SAb**	0.26_2,17_	ns	0.09_2,17_	ns	0.80_1,17_	ns	2.84_1,17_	ns	1.04_1,17_	ns
**Cortex**	**ACC**	0.25_2,17_	ns	0.49_2,17_	ns	1.15_1,17_	ns	1.20_1,17_	ns	0.06_1,17_	ns
	**MAC**	0.53_2,17_	ns	0.61_2,17_	ns	0.00_1,17_	ns	3.86_1,17_	ns	0.05_1,17_	ns
	**MC**	0.22_2,17_	ns	1.02_2,17_	ns	1.32_1,17_	ns	8.56_1,17_	**0.009**	1.00_1,17_	ns
	**SS**	0.24_2,17_	ns	0.75_2,17_	ns	1.55_1,17_	ns	7.63_1,17_	**0.013**	0.86_1,17_	ns
	**IC**	0.29_2,17_	ns	0.52_2,17_	ns	1.10_1,17_	ns	4.46_1,17_	**0.007**	6.32_1,17_	**0.022**

**Table 2:** Dose and time-independent effects on haloperidol-mediated *Homer1a* expression as assessed by univariate analysis in each region of interest. On the right side of the table, the “dose effect” was analyzed separately for acute and chronic administration regimens, in order to determine if haloperidol-mediated *Homer1a* expression was different for groups exposed to different haloperidol doses (i.e., HAL0.25, HAL0.5, and HAL0.8). On the left side of the table, the “time effect” was analyzed separately for each haloperidol dose in order to determine if haloperidol-mediated *Homer1a* expression was different for groups with different durations of treatment (i.e., acute and chronic). Significant effects are given in bold. HAL0.25 = haloperidol at 0.25 mg/kg; HAL0.5 = haloperidol at 0.5 mg/kg; HAL0.8 = haloperidol at 0.8 mg/kg. ns = not significant.

		Dose Effect						Time Effect			
		Acute HAL0.25 vs. Acute HAL0.5 vs. Acute HAL0.8		Chronic HAL0.25 vs. Chronic HAL0.5 vs. Chronic HAL0.8		Acute HAL0.25 vs. Chronic HAL0.25		Acute HAL0.5 vs. Chronic HAL0.5		Acute HAL0.8 vs. Chronic HAL0.8	
		F_df_	*p*	F_df_	*p*	F_df_	*p*	F_df_	*p*	F_df_	*p*
**Striatum**	**DM**	4.04_2,16_	**0.038**	0.43_2, 16_	ns	11.40_1,16_	**0.004**	28.33_1,16_	**0.0005**	14.60_1,16_	**0.002**
	**DL**	1.17_2, 16_	ns	0.07_2, 16_	ns	16.18_1,16_	**0.001**	24.50_1,16_	**0.0005**	17.02_1,16_	**0.001**
	**VM**	3.37_2, 16_	ns	0.88_2, 16_	ns	19.90_1,16_	**0.0005**	39.80_1,16_	**0.0005**	26.74_1,16_	**0.0005**
	**VL**	1.44_2, 16_	ns	0.59_2, 16_	ns	13.35_1,16_	**0.002**	16.47_1,16_	**0.001**	12.91_1,16_	**0.002**
	**CAb**	3.34_2, 16_	ns	0.31_2, 16_	ns	14.30_1,16_	**0.002**	33.84_1,16_	**0.0005**	22.26_1,16_	**0.0005**
	**SAb**	4.20_2, 16_	**0.034**	0.40_2, 16_	ns	2.04_1, 16_	ns	19.33_1,16_	**0.0005**	11.89_1,16_	**0.003**
**Cortex**	**ACC**	4.4_2,16_	**0.03**	3.08_2,16_	ns	0.48_1,16_	ns	0.04_1,16_	ns	0.32_1,16_	ns
	**MAC**	1.6_2,16_	ns	0.87_2,16_	ns	1.54_1,16_	ns	3.11_1,16_	ns	2.19_1,16_	ns
	**MC**	3.17_2,16_	ns	0.74_2,16_	ns	1.76_1,16_	ns	7.59_1,16_	**0.014**	10.39_1,16_	**0.005**
	**SS**	2.13_2,16_	ns	0.70_2,16_	ns	0.70_1,16_	ns	0.69_1,16_	ns	8.03_1,16_	**0.021**
	**IC**	1.39_2,16_	ns	0.82_2,16_	ns	3.37_1,16_	ns	3.48_1,16_	ns	13.67_1,16_	**0.002**

**Table 3:** Treatment and time-independent effects of different receptor profile antipsychotics on *Homer1a* expression as assessed by univariate analysis in each region of interest. The treatment effect was analyzed separately for acute and chronic administration regimens. The time effect was analyzed separately for each antipsychotic treatment group (i.e., OLA2.5, ASE0.1, and HAL0.5). Significant effects are given in bold. OLA2.5 = olanzapine at 2.5 mg/kg; ASE0.1 = asenapine at 0.1 mg/kg; HAL0.5 = haloperidol at 0.5 mg/kg. ns = not significant.

		Treatment Effect				Time Effect					
		Acute OLA2.5 vs. Acute ASE0.1 vs. Acute HAL0.5		Chronic OLA2.5 vs. Chronic ASE0.1 vs. Chronic HAL0.5		Acute OLA2.5 vs. Chronic OLA2.5		Acute ASE0.1 vs. Chronic ASE0.1		Acute HAL0.5 vs. Chronic HAL0.5	
		F_df_	*p*	F_df_	*p*	F_df_	*p*	F_df_	*p*	F_df_	*p*
**Striatum**	**DM**	5.45_2,15_	**0.017**	0.76_2,15_	ns	1.96_1,15_	ns	12.14_1,17_	**0.003**	28.33_1,16_	**0.0005**
	**DL**	3.00_2,15_	ns	0.50_2,15_	ns	3.86_1,15_	ns	21.07_1,17_	**0.0005**	24.50_1,16_	**0.0005**
	**VM**	5.75_2,15_	**0.014**	0.20_2,15_	ns	4.47_1,15_	**0.05**	15.81_1,17_	**0.001**	39.80_1,16_	**0.0005**
	**VL**	4.91_2,15_	**0.023**	1.15_2,15_	ns	1.68_1,15_	ns	10.17_1,17_	**0.005**	16.47_1,16_	**0.001**
	**CAb**	5.65_2,15_	**0.015**	0.25_2,15_	ns	2.86_1,15_	ns	11.32_1,17_	**0.004**	33.84_1,16_	**0.0005**
	**SAb**	2.23_2,15_	ns	0.18_2,15_	ns	0.05_1,15_	ns	2.84_1,17_	ns	19.33_1,16_	**0.0005**
**Cortex**	**ACC**	0.99_2,15_	ns	1.57_2,15_	ns	0.00_1,15_	ns	1.20_1,17_	ns	0.04_1,16_	ns
	**MAC**	1.55_2,15_	ns	0.36_2,15_	ns	0.02_1,15_	ns	3.86_1,17_	ns	3.11_1,16_	ns
	**MC**	0.90_2,15_	ns	0.69_2,15_	ns	0.42_1,15_	ns	8.56_1,17_	**0.009**	7.59_1,16_	**0.014**
	**SS**	0.84_2,15_	ns	1.20_2,15_	ns	0.05_1,15_	ns	7.63_1,17_	**0.013**	0.69_1,16_	ns
	**IC**	0.15_2,15_	ns	1.25_2,15_	ns	2.68_1,15_	ns	4.46_1,17_	**0.007**	3.48_1,16_	ns

**Table 4:** Overall output of multivariate statistical analyses. In this table, we report the overall effects of multivariate statistical analyses carried out in the present study. Three-way interaction reported the effect of ROI*dose/treatment*time. Two-way effects were subdivided in within subjects (i.e., ROI*dose/treatment and ROI*time) and between subjects (i.e., dose/treatment*time). Bold highlight indicates significant effect. ROI = Regions of Interest; ASE = asenapine; HAL = haloperidol; DRPA = different receptor profile antipsychotics.

	Three-Way Interaction	Two-Way Within-Subject (ROI*Dose/Treatment)	Two-Way Within-Subject (ROI*Time)	Two-Way Between-Subject (Dose/Treatment*Time)
**ASE Striatum**	**F_10,85_ = 1.99**	**F_10,85_ = 2.46**	**F_5,85_ = 11.66**	F_2,17_ = 0.53
	***P* = 0.04**	***P* = 0.012**	***P* < 0.0005**	*P* > 0.05
**ASE** **Cortex**	F_8,68_ = 1.31	F_8,68_ = 0.29	**F_4,68_ = 12.42**	F_2,17_ = 0.81
	*P* > 0.05	*P* > 0.05	***P* < 0.0005**	*P* > 0.05
**HAL Striatum**	F_10,80_ = 0.51	F_10,80_ = 0.57	**F_5,80_ = 12.74**	F_2,16_ = 0.81
	*P* > 0.05	*P* > 0.05	***P* < 0.0005**	*P* > 0.05
**HAL** **Cortex**	F_8,64_ = 1.23	F_8,64_ = 1.52	**F_4,64_ = 13.02**	F_2,16_ = 0.57
	*P* > 0.05	*P* > 0.05	***P* < 0.0005**	*P* > 0.05
**DRPA Striatum**	F_10,75_ = 0.19	F_10,75_ = 1.44	**F_5,75_ = 8.64**	F_2,15_ = 1.83
	*P* > 0.05	*P* > 0.05	***P* < 0.0005**	*P* >0.05
**DRPA Cortex**	F_8,60_ = 1.31	F_8,60_ = 1.19	**F_4,60_ = 9.17**	F_2,15_ = 0.48
	*P* > 0.05	*P* > 0.05	***P* < 0.0005**	*P* > 0.05

## Supplementary Materials

Supplementary materials can be found at https://www.mdpi.com/1422-0067/21/15/5555/s1.
