# Supplementary material for: The Effects of Antipsychotics on the Synaptic Plasticity Gene Homer1a Depend on a Combination of Their Receptor Profile, Dose, Duration of Treatment, and Brain Regions Targeted
Source: Int J Mol Sci. 2020 Aug 3;21(15):5555. doi: 10.3390/ijms21155555 (PMC7432375; doi:10.3390/ijms21155555)
Supplement: Supplementary file 1 [file ijms-21-05555-s001.pdf]

Paradigm 1	Acute Treatments	Normalized Data	Raw Data
ACC	Asenapine 0.05mg/kg	p=0.384	p=0.384
	Asenapine 0.1mg/kg	p=0.300	p=0.300
	Asenapine 0.3mg/kg	p=0.119	p=0.119
	Vehicle		p=0.885
MAC	Asenapine 0.05mg/kg	p=0.386	p=0.386
	Asenapine 0.1mg/kg	p=0.276	p=0.276
	Asenapine 0.3mg/kg	p=0.207	p=0.207
	Vehicle		p=0.055
MC	Asenapine 0.05mg/kg	p=0.931	p=0.931
	Asenapine 0.1mg/kg	p=0.181	p=0.181
	Asenapine 0.3mg/kg	p=0.174	p=0.174
	Vehicle		p=0.636
SS	Asenapine 0.05mg/kg	p=0.972	p=0.972
	Asenapine 0.1mg/kg	p=0.113	p=0.113
	Asenapine 0.3mg/kg	p=0.326	p=0.326
	Vehicle		p=0.737
IC	Asenapine 0.05mg/kg	p=0.350	p=0.350
	Asenapine 0.1mg/kg	p=0.397	p=0.397
	Asenapine 0.3mg/kg	p=0.305	p=0.305
	Vehicle		p=0.389
DM	Asenapine 0.05mg/kg	p=0.194	p=0.194
	Asenapine 0.1mg/kg	p=0.190	p=0.190
	Asenapine 0.3mg/kg	p=0.865	p=0.865
	Vehicle		p=0.820
DL	Asenapine 0.05mg/kg	p=0.944	p=0.944
	Asenapine 0.1mg/kg	p=0.400	p=0.400
	Asenapine 0.3mg/kg	p=0.712	p=0.712
	Vehicle		p=0.016
VM	Asenapine 0.05mg/kg	p=0.308	p=0.308
	Asenapine 0.1mg/kg	p=0.054	p=0.054
	Asenapine 0.3mg/kg	p=0.357	p=0.357
	Vehicle		p=0.990
VL	Asenapine 0.05mg/kg	p=0.549	p=0.549
	Asenapine 0.1mg/kg	p=0.218	p=0.218
	Asenapine 0.3mg/kg	p=0.754	p=0.754
	Vehicle		p=0.002
CAb	Asenapine 0.05mg/kg	p=0.846	p=0.846
	Asenapine 0.1mg/kg	p=0.356	p=0.356
	Asenapine 0.3mg/kg	p=0.880	p=0.880
	Vehicle		p=0.741
SAb	Asenapine 0.05mg/kg	p=0.515	p=0.515
	Asenapine 0.1mg/kg	p=0.638	p=0.638
	Asenapine 0.3mg/kg	p=0.188	p=0.188
	Vehicle		p=0.290

Table S1. Outputs of Shapiro-Wilk test for normality in Paradigm 1 acute treatment groups.

Paradigm 1	Chronic Treatments	Normalized Data	Raw Data
ACC	Asenapine 0.05mg/kg	p=0.829	p=0.829
	Asenapine 0.1mg/kg	p=0.330	p=0.330
	Asenapine 0.3mg/kg	p=0.433	p=0.433
	Vehicle		p=0.266
MAC	Asenapine 0.05mg/kg	p=0.289	p=0.289
	Asenapine 0.1mg/kg	p=0.560	p=0.560
	Asenapine 0.3mg/kg	p=0.077	p=0.077
	Vehicle		p=0.946
MC	Asenapine 0.05mg/kg	p=0.610	p=0.610
	Asenapine 0.1mg/kg	p=0.224	p=0.224
	Asenapine 0.3mg/kg	p=0.224	p=0.224
	Vehicle		p=0.322
SS	Asenapine 0.05mg/kg	p=0.436	p=0.436
	Asenapine 0.1mg/kg	p=0.815	p=0.815
	Asenapine 0.3mg/kg	p=0.762	p=0.762
	Vehicle		p=0.997
IC	Asenapine 0.05mg/kg	p=0.314	p=0.314
	Asenapine 0.1mg/kg	p=0.347	p=0.347
	Asenapine 0.3mg/kg	p=0.500	p=0.500
	Vehicle		p=0.318
DM	Asenapine 0.05mg/kg	p=0.359	p=0.359
	Asenapine 0.1mg/kg	p=0.907	p=0.907
	Asenapine 0.3mg/kg	p=0.993	p=0.993
	Vehicle		p=0.311
DL	Asenapine 0.05mg/kg	p=0.327	p=0.327
	Asenapine 0.1mg/kg	p=0.869	p=0.869
	Asenapine 0.3mg/kg	p=0.245	p=0.245
	Vehicle		p=0.292
VM	Asenapine 0.05mg/kg	p=0.136	p=0.136
	Asenapine 0.1mg/kg	p=0.508	p=0.508
	Asenapine 0.3mg/kg	p=0.493	p=0.493
	Vehicle		p=0.487
VL	Asenapine 0.05mg/kg	p=0.058	p=0.058
	Asenapine 0.1mg/kg	p=0.410	p=0.410
	Asenapine 0.3mg/kg	p=0.311	p=0.311
	Vehicle		p=0.416
CAb	Asenapine 0.05mg/kg	p=0.424	p=0.424
	Asenapine 0.1mg/kg	p=0.438	p=0.438
	Asenapine 0.3mg/kg	p=0.263	p=0.263
	Vehicle		p=0.253
SAb	Asenapine 0.05mg/kg	p=0.873	p=0.873
	Asenapine 0.1mg/kg	p=0.294	p=0.294
	Asenapine 0.3mg/kg	p=0.434	p=0.434
	Vehicle		p=0.734

Table S2. Outputs of Shapiro-Wilk test for normality in Paradigm 1 chronic treatment groups.

Paradigm 2	Acute Treatments	Normalized Data	Raw Data
ACC	Haloperidol 0.25mg/kg	p=0.105	p=0.105
	Haloperidol 0.5mg/kg	p=0.136	p=0.136
	Haloperidol 0.8mg/kg	p=0.819	p=0.819
	Vehicle		p=0.885
MAC	Haloperidol 0.25mg/kg	p=0.996	p=0.996
	Haloperidol 0.5mg/kg	p=0.875	p=0.875
	Haloperidol 0.8mg/kg	p=0.408	p=0.408
	Vehicle		p=0.055
MC	Haloperidol 0.25mg/kg	p=0.825	p=0.825
	Haloperidol 0.5mg/kg	p=0.986	p=0.986
	Haloperidol 0.8mg/kg	p=0.914	p=0.914
	Vehicle		p=0.636
SS	Haloperidol 0.25mg/kg	p=0.694	p=0.694
	Haloperidol 0.5mg/kg	p=0.184	p=0.184
	Haloperidol 0.8mg/kg	p=0.373	p=0.373
	Vehicle		p=0.737
IC	Haloperidol 0.25mg/kg	p=0.164	p=0.164
	Haloperidol 0.5mg/kg	p=0.965	p=0.965
	Haloperidol 0.8mg/kg	p=0.792	p=0.792
	Vehicle		p=0.389
DM	Haloperidol 0.25mg/kg	p=0.463	p=0.463
	Haloperidol 0.5mg/kg	p=0.315	p=0.315
	Haloperidol 0.8mg/kg	p=0.890	p=0.890
	Vehicle		p=0.820
DL	Haloperidol 0.25mg/kg	p=0.01	p=0.01
	Haloperidol 0.5mg/kg	p=0.654	p=0.654
	Haloperidol 0.8mg/kg	p=0.01	p=0.01
	Vehicle		p=0.01
VM	Haloperidol 0.25mg/kg	p=0.232	p=0.232
	Haloperidol 0.5mg/kg	p=0.363	p=0.363
	Haloperidol 0.8mg/kg	p=0.124	p=0.124
	Vehicle		p=0.990
VL	Haloperidol 0.25mg/kg	p=0.679	p=0.679
	Haloperidol 0.5mg/kg	p=0.308	p=0.308
	Haloperidol 0.8mg/kg	p=0.083	p=0.083
	Vehicle		p=0.002
CAb	Haloperidol 0.25mg/kg	p=0.068	p=0.068
	Haloperidol 0.5mg/kg	p=0.376	p=0.376
	Haloperidol 0.8mg/kg	p=0.403	p=0.403
	Vehicle		p=0.741
SAb	Haloperidol 0.25mg/kg	p=0.142	p=0.142
	Haloperidol 0.5mg/kg	p=0.271	p=0.271
	Haloperidol 0.8mg/kg	p=0.049	p=0.049
	Vehicle		p=0.290

Table S3. Outputs of Shapiro-Wilk test for normality in Paradigm 2 acute treatment groups.

Paradigm 2	Chronic Treatments	Normalized Data	Raw Data
ACC	Haloperidol 0.25mg/kg	p=0.639	p=0.639
	Haloperidol 0.5mg/kg	p=0.122	p=0.122
	Haloperidol 0.8mg/kg	p=0.802	p=0.802
	Vehicle		p=0.266
MAC	Haloperidol 0.25mg/kg	p=0.703	p=0.703
	Haloperidol 0.5mg/kg	p=0.963	p=0.963
	Haloperidol 0.8mg/kg	p=0.349	p=0.349
	Vehicle		p=0.946
MC	Haloperidol 0.25mg/kg	p=0.063	p=0.063
	Haloperidol 0.5mg/kg	p=0.296	p=0.296
	Haloperidol 0.8mg/kg	p=0.848	p=0.848
	Vehicle		p=0.322
SS	Haloperidol 0.25mg/kg	p=0.721	p=0.721
	Haloperidol 0.5mg/kg	p=0.109	p=0.109
	Haloperidol 0.8mg/kg	p=0.253	p=0.253
	Vehicle		p=0.997
IC	Haloperidol 0.25mg/kg	p=0.042	p=0.042
	Haloperidol 0.5mg/kg	p=0.710	p=0.710
	Haloperidol 0.8mg/kg	p=0.253	p=0.253
	Vehicle		p=0.318
DM	Haloperidol 0.25mg/kg	p=0.675	p=0.675
	Haloperidol 0.5mg/kg	p=0.898	p=0.898
	Haloperidol 0.8mg/kg	p=0.971	p=0.971
	Vehicle		p=0.311
DL	Haloperidol 0.25mg/kg	p=0.590	p=0.590
	Haloperidol 0.5mg/kg	p=0.489	p=0.489
	Haloperidol 0.8mg/kg	p=0.903	p=0.903
	Vehicle		p=0.292
VM	Haloperidol 0.25mg/kg	p=0.829	p=0.829
	Haloperidol 0.5mg/kg	p=0.724	p=0.724
	Haloperidol 0.8mg/kg	p=0.317	p=0.317
	Vehicle		p=0.487
VL	Haloperidol 0.25mg/kg	p=0.805	p=0.805
	Haloperidol 0.5mg/kg	p=0.923	p=0.923
	Haloperidol 0.8mg/kg	p=0.572	p=0.572
	Vehicle		p=0.416
CAb	Haloperidol 0.25mg/kg	p=0.142	p=0.142
	Haloperidol 0.5mg/kg	p=0.171	p=0.171
	Haloperidol 0.8mg/kg	p=0.002	p=0.002
	Vehicle		p=0.252
SAb	Haloperidol 0.25mg/kg	p=0.968	p=0.968
	Haloperidol 0.5mg/kg	p=0.023	p=0.023
	Haloperidol 0.8mg/kg	p=0.194	p=0.194
	Vehicle		p=0.734

Table S4. Outputs of Shapiro-Wilk test for normality in Paradigm 2 chronic treatment groups.

Paradigm 3	Acute Treatments	Normalized Data	Raw Data
ACC	Olanzapine 2.5mg/kg	p=0.164	p=0.164
	Asenapine 0.1mg/kg	p=0.776	p=0.776
	Haloperidol 0.5mg/kg	p=0.436	p=0.436
	Vehicle		p=0.078
MAC	Olanzapine 2.5mg/kg	p=0.926	p=0.926
	Asenapine 0.1mg/kg	p=0.338	p=0.338
	Haloperidol 0.5mg/kg	p=0.723	p=0.723
	Vehicle		p=0.623
MC	Olanzapine 2.5mg/kg	p=0.203	p=0.203
	Asenapine 0.1mg/kg	p=0.661	p=0.661
	Haloperidol 0.5mg/kg	p=0.589	p=0.589
	Vehicle		p=0.478
SS	Olanzapine 2.5mg/kg	p=0.355	p=0.355
	Asenapine 0.1mg/kg	p=0.521	p=0.521
	Haloperidol 0.5mg/kg	p=0.127	p=0.127
	Vehicle		p=0.982
IC	Olanzapine 2.5mg/kg	p=0.704	p=0.704
	Asenapine 0.1mg/kg	p=0.773	p=0.773
	Haloperidol 0.5mg/kg	p=0.234	p=0.234
	Vehicle		p=0.023
DM	Olanzapine 2.5mg/kg	p=0.831	p=0.831
	Asenapine 0.1mg/kg	p=0.667	p=0.667
	Haloperidol 0.5mg/kg	p=0.521	p=0.521
	Vehicle		p=0.148
DL	Olanzapine 2.5mg/kg	p=0.061	p=0.061
	Asenapine 0.1mg/kg	p=0.803	p=0.803
	Haloperidol 0.5mg/kg	p=0.640	p=0.640
	Vehicle		p=0.669
VM	Olanzapine 2.5mg/kg	p=0.988	p=0.988
	Asenapine 0.1mg/kg	p=0.441	p=0.441
	Haloperidol 0.5mg/kg	p=0.169	p=0.169
	Vehicle		p=0.639
VL	Olanzapine 2.5mg/kg	p=0.787	p=0.787
	Asenapine 0.1mg/kg	p=0.883	p=0.883
	Haloperidol 0.5mg/kg	p=0.660	p=0.660
	Vehicle		p=0.529
CAb	Olanzapine 2.5mg/kg	p=0.227	p=0.227
	Asenapine 0.1mg/kg	p=0.621	p=0.621
	Haloperidol 0.5mg/kg	p=0.389	p=0.389
	Vehicle		p=0.889
SAb	Olanzapine 2.5mg/kg	p=0.370	p=0.370
	Asenapine 0.1mg/kg	p=0.590	p=0.590
	Haloperidol 0.5mg/kg	p=0.427	p=0.427
	Vehicle		p=0.287

Table S5. Outputs of Shapiro-Wilk test for normality in Paradigm 3 acute treatment groups.

Paradigm 3	Chronic Treatments	Normalized Data	Raw Data
ACC	Olanzapine 2.5mg/kg	p=0.365	p=0.365
	Asenapine 0.1mg/kg	p=0.589	p=0.589
	Haloperidol 0.5mg/kg	p=0.250	p=0.250
	Vehicle		p=0.072
MAC	Olanzapine 2.5mg/kg	p=0.663	p=0.663
	Asenapine 0.1mg/kg	p=0.268	p=0.268
	Haloperidol 0.5mg/kg	p=0.170	p=0.170
	Vehicle		p=0.485
MC	Olanzapine 2.5mg/kg	p=0.311	p=0.311
	Asenapine 0.1mg/kg	p=0.328	p=0.328
	Haloperidol 0.5mg/kg	p=0.160	p=0.160
	Vehicle		p=0.059
SS	Olanzapine 2.5mg/kg	p=0.041	p=0.041
	Asenapine 0.1mg/kg	p=0.815	p=0.815
	Haloperidol 0.5mg/kg	p=0.590	p=0.590
	Vehicle		p=0.639
IC	Olanzapine 2.5mg/kg	p=0.623	p=0.623
	Asenapine 0.1mg/kg	p=0.469	p=0.469
	Haloperidol 0.5mg/kg	p=0.160	p=0.160
	Vehicle		p=0.479
DM	Olanzapine 2.5mg/kg	p=0.354	p=0.354
	Asenapine 0.1mg/kg	p=0.759	p=0.759
	Haloperidol 0.5mg/kg	p=0.823	p=0.823
	Vehicle		p=0.278
DL	Olanzapine 2.5mg/kg	p=0.559	p=0.559
	Asenapine 0.1mg/kg	p=0.289	p=0.289
	Haloperidol 0.5mg/kg	p=0.378	p=0.378
	Vehicle		p=0.921
VM	Olanzapine 2.5mg/kg	p=0.367	p=0.367
	Asenapine 0.1mg/kg	p=0.469	p=0.469
	Haloperidol 0.5mg/kg	p=0.278	p=0.278
	Vehicle		p=0.097
VL	Olanzapine 2.5mg/kg	p=0.517	p=0.517
	Asenapine 0.1mg/kg	p=0.589	p=0.589
	Haloperidol 0.5mg/kg	p=0.624	p=0.624
	Vehicle		p=0.480
CAb	Olanzapine 2.5mg/kg	p=0.061	p=0.061
	Asenapine 0.1mg/kg	p=0.378	p=0.378
	Haloperidol 0.5mg/kg	p=0.270	p=0.270
	Vehicle		p=0.945
SAb	Olanzapine 2.5mg/kg	p=0.554	p=0.554
	Asenapine 0.1mg/kg	p=0.628	p=0.628
	Haloperidol 0.5mg/kg	p=0.489	p=0.489
	Vehicle		p=0.834

Table S6. Outputs of Shapiro-Wilk test for normality in Paradigm 3 chronic treatment groups.

	**3way effect**	**2way within-subject (ROI*Dose/Treatment)**	**2way within-subject (ROI*Time)**	**2way between-subject (Time*Dose/Treatment)**	**Univari[illegible] Topogr[illegible]**
**ASE Cortex**	$F_{12,92}=1.28$ $p>0.05$	$F_{12,92}=0.45$ $p>0.05$	$F_{4,92}=1.26$ $p>0.05$	$F_{3,23}=1.46$ $p>0.05$	**$F_{4,92}=71$[illegible] $p<0.000$[illegible]**
**ASE Striatum**	**$F_{15,115}=2.03$ $p=0.018$**	**$F_{15,115}=3.77$ $p<0.0005$**	**$F_{5,115}=4.35$ $p=0.001$**	*$F_{3,23}=2.52$ $p=0.08$*	**$F_{5,115}=1$[illegible] $p<0.000$[illegible]**
**HAL Cortex**	$F_{12,88}=1.29$ $p>0.05$	$F_{12,88}=0.85$ $p>0.05$	$F_{4,88}=1.77$ $p>0.05$	$F_{3,22}=1.45$ $p>0.05$	**$F_{4,88}=40$[illegible] $p<0.000$[illegible]**
**HAL Striatum**	$F_{15,110}=1.39$ $p>0.05$	**$F_{15,110}=3.46$ $p<0.0005$**	**$F_{5,110}=4.71$ $p=0.001$**	**$F_{3,22}=6.84$ $p=0.002$**	**$F_{5,110}=1$[illegible] $p<0.000$[illegible]**
**DRPA Cortex**	$F_{12,84}=1.15$ $p>0.05$	$F_{12,84}=0.65$ $p>0.05$	*$F_{4,84}=2.16$ $p=0.08$*	$F_{3,21}=1.503$ $p>0.05$	**$F_{4,84}=50$[illegible] $p<0.000$[illegible]**
**DRPA Striatum**	$F_{15,100}=0.75$ $p>0.05$	*$F_{15,100}=1.63$ $p=0.08$*	$F_{5,100}=1.1$ $p>0.05$	**$F_{3,20}=3.74$ $p=0.03$**	**$F_{5,100}=4$[illegible] $p=0.00$[illegible]**

Table S7. Overall statistical analyses of raw data, i.e. including the vehicle group.

	**2way (Time*Dose)**	**Pairwise Time**	**Pairwise Dose**
**ACC**	$F_{3,23}$=0.36, p>0.05	ns	ns
**MAC**	$F_{3,23}$=1.05, p>0.05	ns	ns
**MC**	$F_{3,23}$=1.98, p>0.05	ns	ns
**SS**	$F_{3,23}$=1.75, p>0.05	ns	ns
**IC**	$F_{3,23}$=2.27, p>0.05	VEH: Acute < Chronic*	ns
**DM**	$F_{3,23}$=2.28, p>0.05	ASE0.1: Acute > Chronic*	ns
**DL**	**$F_{3,23}$=4.61, p=0.012**	ASE0.1: Acute > Chronic*<br>*ASE0.3: Acute > Chronic*<br>VEH: Acute < Chronic*	Acute: ASE0.05, ASE0.1, ASE0.3 > VEH**
**VM**	**$F_{3,23}$=3.19, p=0.04**	VEH: Acute < Chronic*	ns
**VL**	$F_{3,23}$=2.31, p>0.05	ASE0.1: Acute > Chronic*	ns
**CAb**	$F_{3,23}$=2.32, p>0.05	VEH: Acute < Chronic*	Acute: ASE0.1 > VEH*
**SAb**	$F_{3,23}$=0.61, p>0.05	ns	ns

Table S8. Dose and Time combined and independent effects on asenapine-mediated Homer1a expression. Data were given as raw data, i.e. including the vehicle group for multiple comparisons. *: p<0.05; **: p<0.01

	2way (Time*Dose)	Pairwise Time	Pairwise Dose
**ACC**	$F_{3,22}$=0.22, p>0.05	ns	ns
**MAC**	$F_{3,22}$=0.59, p>0.05	ns	ns
**MC**	$F_{3,22}$=2.39, p>0.05	VEH: Acute < Chronic*	ns
**SS**	$F_{3,22}$=1.48, p>0.05	VEH: Acute < Chronic*	ns
**IC**	$F_{3,22}$=2.26, p>0.05	VEH: Acute < Chronic**	ns
**DM**	**$F_{3,22}$=4.74, p=0.01**	HAL0.5: Acute > Chronic**	Acute: HAL0.25 > VEH*<br>Acute: HAL0.5 > VEH**
**DL**	**$F_{3,22}$=6.006, p=0.004**	HAL0.25: Acute > Chronic*<br>HAL0.5: Acute > Chronic**<br>HAL0.8: Acute > Chronic*<br>*VEH: Acute < Chronic*[a]	Acute: HAL0.25, HAL0.5, HAL0.8 > VEH**
**VM**	**$F_{3,22}$=7.29, p=0.001**	HAL0.5: Acute > Chronic**<br>HAL0.8: Acute > Chronic*<br>VEH: Acute < Chronic*	Acute: HAL0.25, HAL0.5 > VEH**<br>Acute: HAL0.8 > VEH*
**VL**	**$F_{3,22}$=4.25, p=0.01**	HAL0.25: Acute > Chronic*<br>HAL0.5: Acute > Chronic**<br>HAL0.8: Acute > Chronic*	Acute: HAL0.25, HAL0.5, HAL0.8 > VEH**
**CAb**	**$F_{3,22}$=6.95, p=0.002**	HAL0.5: Acute > Chronic**<br>HAL0.8: Acute > Chronic*<br>VEH: Acute < Chronic*	Acute: HAL0.25, HAL0.5, HAL0.8 > VEH**
**SAb**	**$F_{3,22}$=4.34, p=0.01**	HAL0.5: Acute > Chronic*	Acute: HAL0.5 > VEH**<br>Acute: HAL0.8 > VEH*<br>Acute: HAL0.5 > HAL0.25*

Table S9. Dose and Time combined and independent effects on haloperidol-mediated Homer1a expression. Data were given as raw data, i.e. including the vehicle group for multiple comparisons. *: p<0.05; **: p<0.01; a: trend toward significance.

	2way (Time*Dose)	Pairwise Time	Pairwise Dose
**ACC**	$F_{3,21}$=0.34, p>0.05	ns	ns
**MAC**	$F_{3,21}$=1.004, p>0.05	ns	ns
**MC**	$F_{3,21}$=2.09, p>0.05	*VEH: Acute < Chronic*[a]	ns
**SS**	$F_{3,21}$=1.92, p>0.05	ns	ns
**IC**	$F_{3,21}$=1.71, p>0.05	VEH: Acute < Chronic*	ns
**DM**	**$F_{3,21}$=4.51, p=0.014**	HAL0.5: Acute > Chronic**<br>ASE0.1: Acute > Chronic*	Acute: HAL0.5 > VEH**<br>Acute: HAL0.5 > OLA*
**DL**	**$F_{3,21}$=4.92, p=0.01**	HAL0.5: Acute > Chronic**<br>ASE0.1: Acute > Chronic*	Acute: HAL0.5 > VEH***<br>Acute: ASE0.1 > VEH**
**VM**	**$F_{3,21}$=5.23, p=0.007**	HAL0.5: Acute > Chronic**<br>VEH: Acute < Chronic*	Acute: HAL0.5 > VEH**<br>Acute: HAL0.5 > OLA*
**VL**	**$F_{3,21}$=3.16, p=0.04**	HAL0.5: Acute > Chronic*<br>*ASE0.1: Acute > Chronic*[b]	Acute: HAL0.5 > VEH***<br>Acute: HAL0.5 > OLA*
**CAb**	**$F_{3,21}$=4.86, p=0.01**	HAL0.5: Acute > Chronic**<br>VEH: Acute < Chronic*	Acute: HAL0.5 > VEH***<br>Acute: ASE0.1 > VEH*<br>Acute: HAL0.5 > OLA*
**SAb**	$F_{3,21}$=0.88, p>0.05	ns	ns

Table S10. Dose and Time combined and independent effects on Homer1a expression by different receptor profile antipsychotics. Data were given as raw data, i.e. including the vehicle group for multiple comparisons. *: p<0.05; **: p<0.01; ***: p<0.0005; a: p=0.06; b: p=0.08
